# The Molecular Fingerprint of Dorsal Root and Trigeminal Ganglion Neurons

**DOI:** 10.3389/fnmol.2017.00304

**Published:** 2017-09-26

**Authors:** Douglas M. Lopes, Franziska Denk, Stephen B. McMahon

**Affiliations:** Neurorestoration Group, Wolfson Centre for Age-Related Diseases, King’s College London, London, United Kingdom

**Keywords:** DRG, trigeminal ganglia, RNA sequencing, transcriptome, pain, migraine

## Abstract

The dorsal root ganglia (DRG) and trigeminal ganglia (TG) are clusters of cell bodies of highly specialized sensory neurons which are responsible for relaying information about our environment to the central nervous system. Despite previous efforts to characterize sensory neurons at the molecular level, it is still unknown whether those present in DRG and TG have distinct expression profiles and therefore a unique molecular fingerprint. To address this question, we isolated lumbar DRG and TG neurons using fluorescence-activated cell sorting from Advillin-GFP transgenic mice and performed RNA sequencing. Our transcriptome analyses showed that, despite being overwhelmingly similar, a number of genes are differentially expressed in DRG and TG neurons. Importantly, we identified 24 genes which were uniquely expressed in either ganglia, including an arginine vasopressin receptor and several homeobox genes, giving each population a distinct molecular fingerprint. We compared our findings with published studies to reveal that many genes previously reported to be present in neurons are in fact likely to originate from other cell types in the ganglia. Additionally, our neuron-specific results aligned well with a dataset examining whole human TG and DRG. We propose that the data can both improve our understanding of primary afferent biology and help contribute to the development of drug treatments and gene therapies which seek targets with unique or restricted expression patterns.

## Introduction

Among the different types of cells present in the central (CNS) and peripheral (PNS) nervous systems, sensory neurons are of particular importance as they are continually relaying information about our environment. The trigeminal ganglion (TG) contains many of the sensory neurons innervating the head while the dorsal root ganglia (DRG) mostly innervate the rest of the body. Both groups are highly specialized pseudounipolar neurons that can detect and respond to a variety of chemical, mechanical, and thermal stimuli, serving as a warning system for mammals (Porter and Spencer, 1982; Luo et al., 1991; Capra and Dessem, 1992; Devor, 1999; Woolf and Ma, 2007; Dubin and Patapoutian, 2010). TG neurons, in addition, have some distinct chemosensitive properties relating to olfaction and the gustatory system (Gerhold and Bautista, 2009; Viana, 2011).

DRG and TG are formed by agglomerates of many thousands of primary afferent neurons. There is large heterogeneity, whereby different types of neurons have their own special perceptual modalities and therefore distinct cellular and molecular identities (Snider and McMahon, 1998; Devor, 1999; Marmigere and Ernfors, 2007; Dubin and Patapoutian, 2010; Reichling et al., 2013). Despite their functional similarities and capability to sense innocuous and noxious stimuli, DRG and TG neurons are very distinct in their location and connectivity. DRG afferents are found peripherally alongside the spinal cord and directly synapse onto spinal neurons, whereas TG neurons are localized at the base of the skull, projecting either directly to the brain stem or to the upper regions of the spinal cord (Capra and Dessem, 1992; Erzurumlu et al., 2010). Moreover, DRG neurons derive from the neural crest, whereas TG neurons have dual origin, containing cells originated both from cranial neural crest and trigeminal ectodermal placodes (Altman and Bayer, 1982; D’Amico-Martel and Noden, 1983; Durham and Garrett, 2010; Erzurumlu et al., 2010; Krispin et al., 2010). The distinction in cell fate at embryonic stages, together with some of the already mentioned exclusive functional characteristics and different connectivity patterns, suggests the existence of different molecular identities underlying either of these ganglia.

Studies have indeed started to characterize DRG and TG neurons at the molecular level using RNA sequencing (RNA-seq) (Manteniotis et al., 2013; Chiu et al., 2014; Perkins et al., 2014; Thakur et al., 2014; Flegel et al., 2015; Reynders et al., 2015; Hu et al., 2016; Sapio et al., 2016; Wu et al., 2016; Kogelman et al., 2017) enabling in-depth analysis and characterization of their genome-wide transcriptional profiles. To date a few studies have reported approximately 200 differentially expressed genes in both rodents and humans (Manteniotis et al., 2013; Flegel et al., 2015; Kogelman et al., 2017). Among them were many odorant-binding proteins, ion channels, and G protein-coupled receptors (Manteniotis et al., 2013; Flegel et al., 2015). However, non-neuronal cells account for a significant proportion of cells in these ganglia (Devor, 1999; Hanani, 2005; Durham and Garrett, 2010; Huang et al., 2013) and therefore influence the transcripts present in these datasets, with neuronal genes underrepresented. Thus, a comparison in the expression profiles between enriched neuronal cell populations remains to be performed.

In this study, we examined lumbar DRG and TG ganglia with a specific focus on differences in neuronal gene expression. Using a combination of fluorescence-activated cell sorting (FACS), a transgenic mouse model and RNA-seq analysis, we identified a number of genes exclusively expressed in either DRG or TG neurons. Furthermore, we correlated our data with published studies and reveal that many genes previously believed to be present in neurons, are in fact either expressed in glial cells or other cell types. These findings are not only important for the understanding of the biology of primary afferents as whole, but could also aid development of drug treatments and gene therapies which seek unequivocal targets.

## Results

### Isolation of Specific Populations of Neurons by FACS

**FIGURE 1:**
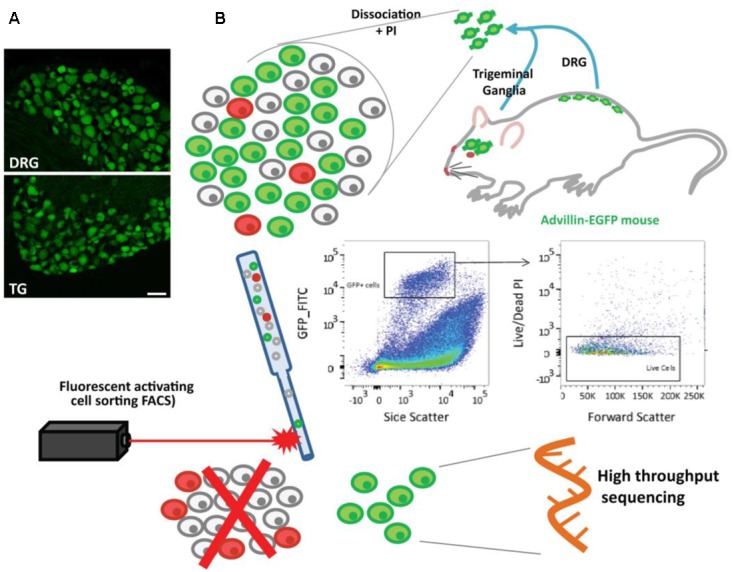
Sensory neurons in the Avil-GFP transgenic mouse and schematic representation of neuronal isolation using flow cytometry. **(A)** Representative picture of DRG and TG ganglion showing the expression of EGFP under the control of the advillin promoter (green cells) in sensory neurons. Scale bar: 50 μm. **(B)** Lumbar DRG and TG were extracted from Avil-EGFP mice. Neurons were dissociated and exposed to propidium iodide (PI), and EGFP+ cells (neurons) were sorted using the gating strategy above, where the non-neuronal cells and PI+ cells were excluded (represented in the cartoon as gray and red). RNA was then extracted and sequenced.

We have previously demonstrated that conventional DRG dissociation results in a mixture of cells in which neurons only account for ∼10% of the population (Thakur et al., 2014). In this study we used a sorting strategy, employing a mouse line expressing GFP under the control of the advillin promotor (Avil-GFP), which labels sensory neurons (Gong et al., 2003) (**Figure 1A** and Supplementary Figure 1). Lumbar DRG and TG were dissociated, the cells were exposed to propidium iodide (PI) and isolated using FACS (**Figure 1B**; for gating strategies, please refer to section “Materials and Methods” and Supplementary Figure 2). From all GFP+/PI- singlets, 500 events were captured for each biological replicate consisting of *n* = 6 mice for DRG and *n* = 4 mice for TG. Culture and analysis of sorted cells revealed that despite limited satellite glial cells, neurons accounted for over 70% of our isolated population (data not shown), a percentage we estimate to be slightly undervalued, given the proliferating nature of non-neuronal cells.

### RNA-Sequencing of DRG and TG Neurons

Following the isolation of neurons by FACS, high-quality RNA was extracted (average RIN 8.75, min 7.6, max 9.9) and RNA-seq was performed on polyA+ mRNA using six DRG and four TG replicates. Between 20.7 and 30.3 million reads were uniquely aligned to the mm10 genome using STAR (Dobin et al., 2013), corresponding to a median mapping percentage of 85.8%. Normalized gene expression values and differential gene expression were generated using the cufflinks and DESeq2 algorithms (Trapnell et al., 2010; Love et al., 2014), respectively. Power calculations (using RNAPower; Hart et al., 2013) indicated that we were well-powered to detect twofold differences in gene expression (71% chance for all genes and 81–87% chance for the top 50% of all expressed genes).

**FIGURE 2:**
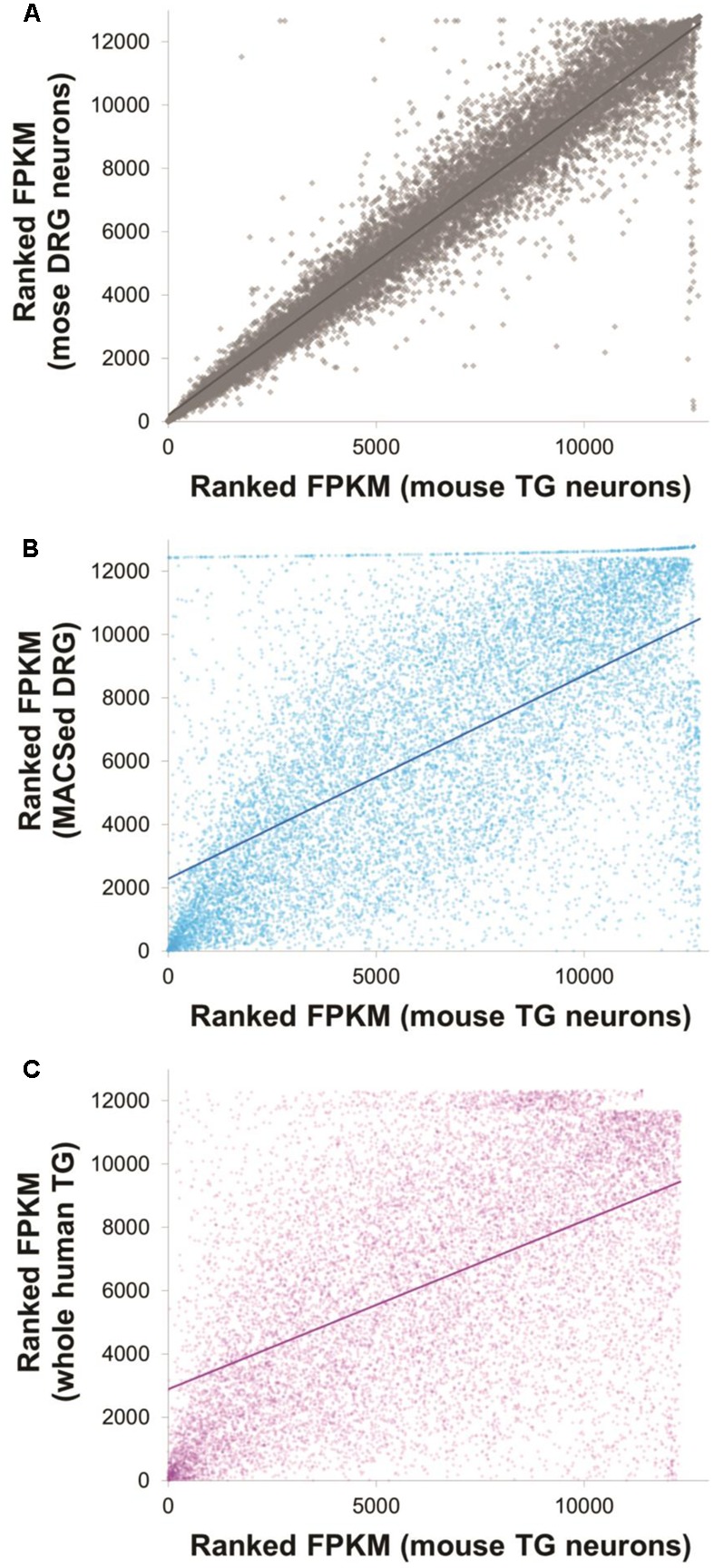
Expression profiles of mouse trigeminal neurons were well correlated with other, previously published datasets. Ranked FPKM values for mouse TG neurons obtained in this dataset are plotted against ranked FPKM values of three different comparison datasets: mouse DRG neurons obtained as part of this particular study (**A**, Spearman’s *r*_s_ = 0.97), magnetically sorted DRG neurons obtained by Thakur et al. (2014) (**B**, Spearman’s *r*_s_ = 0.64) and whole human TG data obtained by Flegel et al. (2015) (**C**, Spearman’s *r*_s_ = 0.53). Solid lines indicate linear correlations. Note that within study correlations are necessarily higher than between study correlations due to the absence of technical batch effects stemming, e.g., from separate library preparation. See Supplementary Table 2 for the underlying data and for additional graphs of presence/absence calls.

A total of 12,638 genes were detected in either DRG or TG neurons (FPKM > 1, in at least two of the replicates for each condition, Supplementary Table 1). Our DRG and TG data were strongly correlated with the ranked expression profiles of pure DRG nociceptors (Thakur et al., 2014): Spearman’s rho *r*_s_ = 0.65, **Figure 2** and Supplementary Table 2. Correlations are very sensitive to library batch effects and further depend on the number and expression levels of the gene sets included in the analysis. We therefore also conducted presence/absence calls, which again showed good overlap: 80% of the top 8000 genes were identified as present in both our current RNA-seq study and the DRG nociceptors dataset (Supplementary Table 2).

**FIGURE 3:**
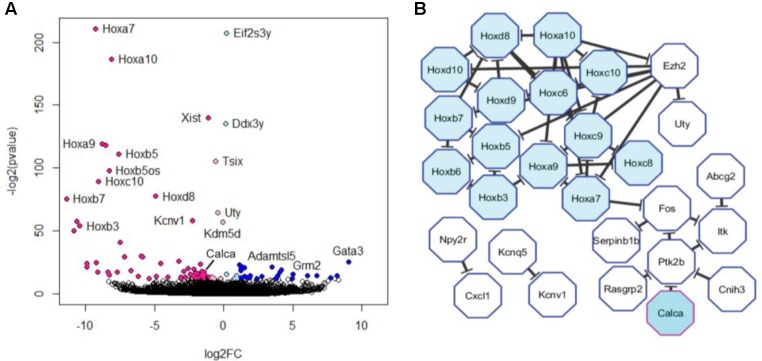
Differential expression of trigeminal versus dorsal root ganglion transcripts. **(A)** Volcano plot of differentially expressed genes obtained using *Deseq2*. Colored dots represent genes that were significantly dysregulated at FDR < 0.05. Genes upregulated in trigeminal ganglion are depicted in light (log2FC > 0) and dark blue (log2FC > 1). Genes upregulated in dorsal root ganglion are depicted in light (log2FC < 0) and dark pink (log2FC < –1). Note that this graph includes genes which are not expressed according to our cut-off as well as those which are enriched in satellite glial cells (see Supplementary Table 1). **(B)** The 44 unambiguously neuronal genes that were found to be upregulated in dorsal root ganglion at FDR < 0.05 were fed into a protein–protein interaction analysis. The resulting network is depicted here and includes 28 genes, 50% of which were Hox genes.

Next, we looked for differences between our DRG and TG samples. DESeq2 analysis revealed 97 genes that were differentially expressed at adjusted *p*-value (adj. *p*) < 0.05 (**Figures 3A,B**), which means that the great majority of genes (more than 99%) were commonly expressed. Of the genes which were different, 44 were upregulated in DRG neurons and 19 were upregulated in TG neurons, with 24 of the genes being present exclusively in either ganglia (**Table 1**). Surprisingly, the vast majority of the latter were homeobox genes (Hox), accounting for ∼50% of the upregulated genes in the DRG (**Figure 3B** and **Table 1**). Further to these neuronal genes, an additional 34 genes emerging from DESeq2 analysis were either not deemed to be expressed according to our FPKM expression cut-off or were not of unambiguously neuronal origin (Supplementary Table 1). The latter was determined by using a previously published RNA-seq dataset (Thakur et al., 2014) in which DRG neurons were magnetically sorted, which prevents any contamination with satellite glial cells (see instruction sheet/tab of Supplementary Table 1 for further information).

**Table 1:** Genes exclusively expressed in either ganglia, identified by RNA sequencing.

		Exclusive expression	
Gene ID	Gene name	DRG	Trigeminal
ENSMUSG00000020123	Avpr1a		✓
ENSMUSG00000038112	AW551984	✓	
ENSMUSG00000063415	Cyp26b1	✓	
ENSMUSG00000029054	Gabrd		✓
ENSMUSG00000078706	Gm53	✓	
ENSMUSG00000043219	Hoxa6	✓	
ENSMUSG00000038236	Hoxa7	✓	
ENSMUSG00000038227	Hoxa9	✓	
ENSMUSG00000000938	Hoxa10	✓	
ENSMUSG00000048763	Hoxb3	✓	
ENSMUSG00000038700	Hoxb5	✓	
ENSMUSG00000000690	Hoxb6	✓	
ENSMUSG00000038721	Hoxb7	✓	
ENSMUSG00000001661	Hoxc6	✓	
ENSMUSG00000001657	Hoxc8	✓	
ENSMUSG00000036139	Hoxc9	✓	
ENSMUSG00000022484	Hoxc10	✓	
ENSMUSG00000027102	Hoxd8	✓	
ENSMUSG00000043342	Hoxd9	✓	
ENSMUSG00000050368	Hoxd10	✓	
ENSMUSG00000028033	Kcnq5	✓	
ENSMUSG00000049112	Oxtr		✓
ENSMUSG00000005268	Prlr	✓	
ENSMUSG00000026475	Rgsl6		✓

*Table listing 24 genes which were exclusively present in the DRG (21 genes) and TG (three genes). First column shows the ENSEMBL Code ID for the gene; the second column lists the gene name and the third/fourth columns display the restrictive expression of the gene either in the DRG or TG (tick marks). Note that the vast majority of the genes are Hox genes. Differential expression was calculated using a Wald test with DESeq2. Exclusivity was considered when FPKM was ≥1 in one neuronal population and not present (FPKM < 1) in the other population, and expression was not depleted in pure neurons (refer to Supplementary Table 1 for additional information)*.

### Validation of Differentially Expressed Genes

**FIGURE 4:**
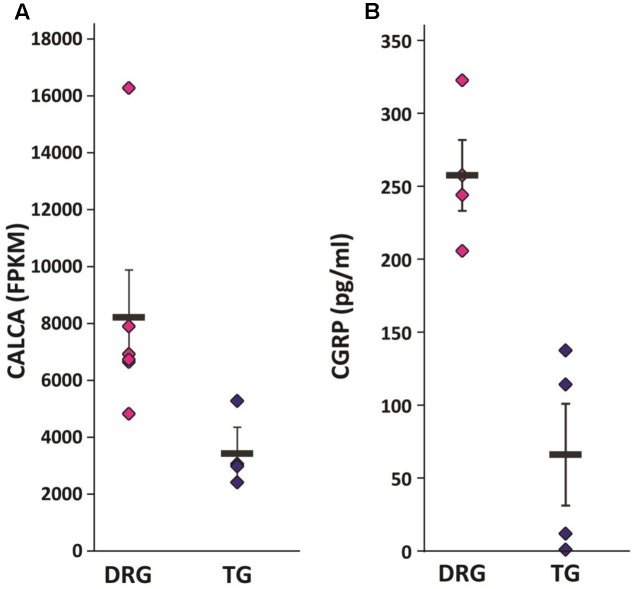
Validation of selected targets. **(A)** CGRP mRNA (Calca) was found to be significantly upregulated in our RNA-seq dataset. Plotted here are individual FPKM values, including means and standard errors of the means. **(B)** CGRP ELISA showed significantly increased protein levels in the DRG versus the TG of a separate cohort of mice: *n* = 4, independent samples *t*-test (two-tailed, heteroscedastic), *p* = 0.005.

**FIGURE 5:**
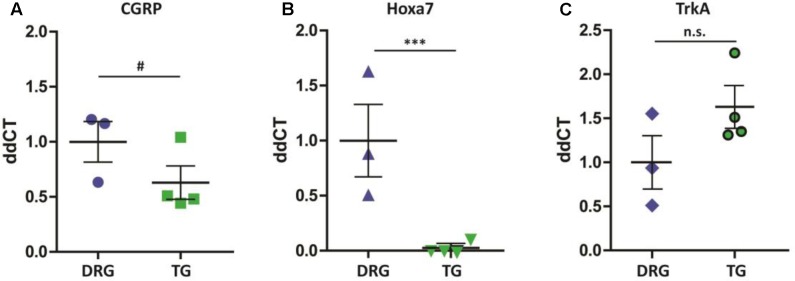
qPCR validation of differentially expressed genes. **(A)** Similarly to the RNA-seq dataset, CGRP mRNA (Calca) was higher in the DRG when compared to TG (^#^*p* = 0.056). **(B)** Hoxa7 gene is exclusively expressed in DRG neurons (^∗∗∗^*p* < 0.001), **(C)** whereas TrkA (Ntrk1) is equally expressed across the two groups of neurons (*p* > 0.05). Results shown are from samples extracted from a separate cohort of mice: *n* = 3 DRG and *n* = 4 TG. Plotted here are individual ddCT values normalized to GAPDH, including means and standard errors of the means; *p*-values were obtained using a Mann–Whitney test.

Given the identification of several differentially expressed genes between DRG and TG neurons in our dataset, we next went on to validate genes with potential functional relevance to pain states. Calcitonin gene-related peptide—CGRP (encoded by the gene CALCA)—is primarily expressed by sensory neurons and known to play a key role in inflammation and neuronal sensitization, particularly during nerve damage (Donnerer and Stein, 1992; Benemei et al., 2009; Yu et al., 2009; Russell et al., 2014) as well as in migraine (Diener et al., 2015; Karsan and Goadsby, 2015; Edvinsson, 2017; Iyengar et al., 2017). Our transcriptome analysis revealed a large difference of approximately 2.5-fold increased expression of the CGRP gene in DRG in relation to TG neurons (AVRG FPKM DRG: 8216 and TG: 3430; adj. *p* < 0.05; **Figure 4A**). To validate this finding we performed a CGRP ELISA from samples extracted from DRG and TG neurons. Results showed that whole DRG has significantly higher level of CGRP protein (**Figure 4B**); indeed, total CGRP levels were over threefold higher when compared to TG (*n* = 4/group; *p* < 0.01; **Figure 4B**). We also carried out qPCR, where we showed a higher amount of CGRP mRNA in the DRG in an independent cohort of animals (*n* = 3 DRG; *n* = 4 TG; *p* = 0.056; **Figure 5A**).

In addition to CGRP, our RNA-seq data also show a differential expression of 15 Hox genes, all of which were exclusively expressed in DRG extracted from the lumbar region. To verify these findings, we performed qPCR in DRG and TG ganglia from a separate cohort of animals. Our results revealed Hoxa7 mRNA to be present only in lumbar DRGs but not in TG neurons (**Figure 5B**, n = 3 DRG; *n* = 4 TG; *p* < 0.01). As a positive internal control, we probed for the Ntrk1 gene (TrkA) and, confirming our RNA-seq analysis, found no difference between the groups (**Figure 5C**).

### Comparison between Mouse and Human Datasets

**FIGURE 6:**
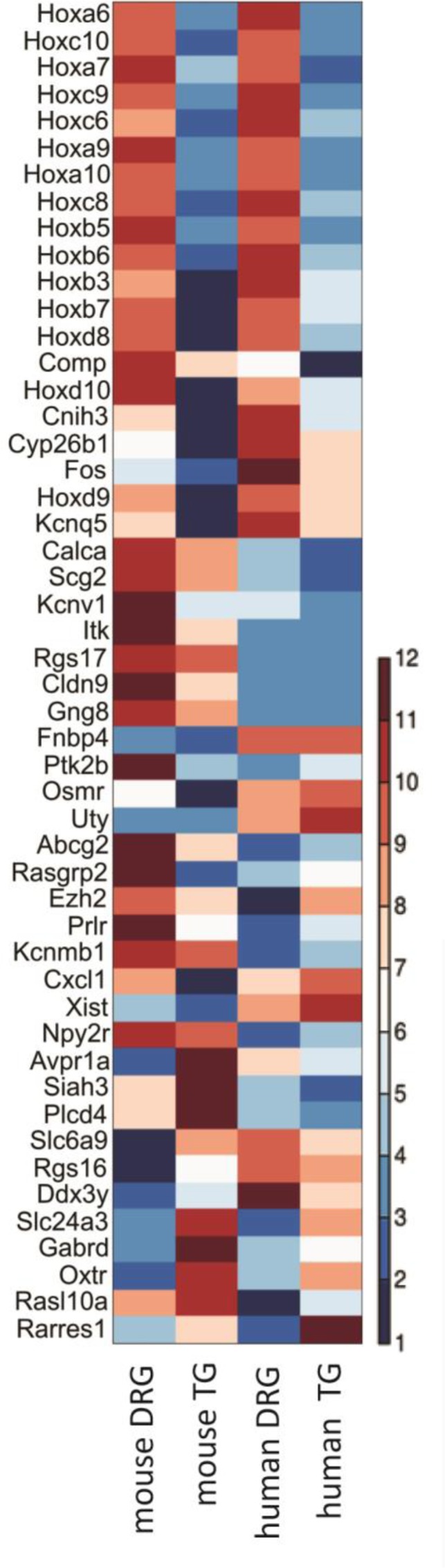
Interspecies comparison of differentially expressed neuronal transcripts in mouse and whole ganglion transcripts in human. Of the 63 genes found to be differentially expressed in DRG and TG, 50 could also be easily identified in a dataset examining expression in human (Flegel et al., 2015). Depicted here is a standardized heatmap of the average log2 (FPKM) values from the two different datasets. Blue indicates negative values, i.e., FPKM < 1, with the genes likely lowly expressed or absent. Increasing levels of expression are represented by increasing degrees of red. See Supplementary Table 3 for the underlying raw FPKM values.

Next, to characterize the similarity between the molecular profiles generated from mouse and human TG, we compared our data to the work of Flegel and collaborators (Flegel et al., 2015). 12,317 genes were found to be expressed in either dataset, 90% of which were identified as equally present or absent in mouse and human (Supplementary Table 2). Rank correlations were moderate (Spearman’s rho *r*_s_ = 0.53, **Figure 2** and Supplementary Table 2). Of the 63 differentially expressed neuronal genes identified in our rodent DRG–TG data, almost 70% followed the same trend toward up- or downregulation in the human data set (**Figure 6** and Supplementary Table 3). Of the genes consistently regulated across species, 21 showed at least a twofold higher FPKM in human DRG compared to TG, while five showed a twofold increase in TG. Together, these analyses demonstrate the validity of our newly generated data and confirm the high molecular similarities between mouse and human sensory ganglia.

## Discussion

Our study is the first to provide a molecular profile of enriched TG and DRG neurons. Isolation of cells by FACS, followed by high throughput sequencing demonstrated key differences in expression level between neuron-specific genes in TG and DRG, among them ion channels as well as genes reportedly involved in pain processing. We validated our results in independent cohorts at message and protein level. Finally, we could demonstrate consistency between our newly generated RNA-seq and a previously published dataset examining whole human TG and DRG.

Studies to date have yielded significant insight into the cell biology of both TG and DRG, giving a clear idea of fiber demography, function, ratio, and distribution of myelinated and non-myelinated fibers in each of these ganglia, as well as how they contribute to nociception, pain, and migraine (Snider and McMahon, 1998; Woolf and Ma, 2007; Dubin and Patapoutian, 2010; DaSilva and DosSantos, 2012; Akerman et al., 2017; Goadsby et al., 2017). However, relatively little is known about the molecular characteristics of these distinct neuronal populations, and how they compare at the transcriptional level. Our data revealed that, despite distinct embryogenic origin and processing capabilities, DRG and TG are surprisingly similar, with an almost identical molecular fingerprint. Nevertheless, we could identify 63 genes which were differentially expressed in these two primary neuron populations, including 24 genes that were found exclusively in either of the ganglia (**Table 1**).

Our results are somewhat unexpected, especially when considering the highly specialized olfactory properties unique to the TG ganglia. Indeed, in a recent study, Manteniotis and collaborators identified almost 200 genes as differentially expressed between whole DRG and TG (Manteniotis et al., 2013). The group described the presence of a number of TG-specific genes and hypothesized their involvement in olfactory processing (Manteniotis et al., 2013). Our results, however, show that none of the genes listed by the authors—including Olfr420, Gm14744, and Lcn3—are expressed by isolated neurons. We believe the contrasting results might be due to tissue composition, given that the majority of cells after DRG dissociation are non-neuronal (Thakur et al., 2014). This can lead to misinterpretation of RNA-seq data: neuron-specific genes could appear falsely downregulated if one type of ganglion were to contain a higher proportion of non-neuronal cells.

Similarly, differences in myelin composition or resident macrophage populations are visible and likely prominent in transcriptional datasets comparing whole DRG and TG. A good example is Kirrel2, which was initially described to be exclusively expressed in TG ganglia (Manteniotis et al., 2013), but is in fact not present in pure TG neurons. Rather, it is highly expressed by astrocytes and oligodendrocytes (Zhang et al., 2014), suggesting differential expression in the satellite glia or myelin of TG and DRG. Similarly, the calcium channels subunits Cacna1g and Cacna1s, which were proposed to be exclusively present in DRG (Manteniotis et al., 2013), are in fact absent from DRG neurons, as shown by our data and previously published work (Thakur et al., 2014; Usoskin et al., 2015). Instead, these genes are found to be expressed in other cells such as microglia and macrophages, both in the CNS and PNS (Gosselin et al., 2014; Lavin et al., 2014; Zhang et al., 2014; Denk et al., 2016), suggesting the non-neuronal origin of these transcripts. Notably, our results and analyses are not disputing the identity of genes previously reported as exclusive to the TG or DRG. We are only providing novel insight into which gene differences are likely to be of neuronal origin. In this context, it is also important to bear in mind that our dissociation method will favor medium and small diameter neurons over large diameter neurons (Supplementary Figure 3). Indeed, genes like Hcn2 and Cacna2d4, which according to single cell data are likely to be exclusively expressed in large diameter DRG neurons (Usoskin et al., 2015; Li et al., 2016), do not pass our FPKM expression cut-off.

The identification of differentially expressed genes allows for the manipulation and targeting of neuronal subpopulations. Here we show a remarkable difference in the expression of the CGRP gene (CALCA) in the two types of ganglia. CGRP is well known for its role as vasodilator and plays a central role in peripheral sensitization, hyperalgesia, and migraine (Benemei et al., 2009; Russell et al., 2014; Iyengar et al., 2017; Schou et al., 2017). Our dataset confirms the presence of CGRP in both TG and DRG neurons, with the latter having over twofold higher expression of this gene—a result which is in line with RNA-seq data derived from human samples (Flegel et al., 2015). These molecular data may seem counterintuitive at first glance. Migraine patients have elevated levels of CGRP (Karsan and Goadsby, 2015; Edvinsson, 2017; Iyengar et al., 2017), the reduction of which has repeatedly been shown to constitute a promising treatment avenue (Olesen et al., 2004; Diener et al., 2015; Karsan and Goadsby, 2015; Mitsikostas and Rapoport, 2015). In contrast, chronic pain conditions involving other areas of the body do not appear to be alleviated by modulation of CGRP (Schou et al., 2017). Yet, DRG appear to express much higher RNA message levels. The discrepancy becomes less jarring when one considers that nerve injury models which cause neuropathic pain conditions are commonly reported to cause downregulation of CGRP (LaCroix-Fralish et al., 2011; Reinhold et al., 2015). This suggests that the gene may be implicated in divergent mechanistic processes in cranial versus peripheral pain conditions.

Another finding from our dataset is the differential regulation of the acid-sensing ion channel subtype-1 (ASIC1), which we identified to have higher expression in TG versus DRG neurons. In addition to having been implicated in neurite outgrowth, neuronal differentiation, synaptic plasticity, learning, memory as well as neuronal injury (Huang et al., 2013; Deval and Lingueglia, 2015; Liu et al., 2017), ASIC1 has a well-documented link to mechanoreception, nociception, and pain (Deval and Lingueglia, 2015; Omerbasic et al., 2015). Previous studies also indicated that ASIC1 could be an analgesic target during pain states (Mazzuca et al., 2007; Diochot et al., 2012). Most notably, ASIC1 has recently been shown to be a new potential drug target for migraine (Holland et al., 2012; Dussor, 2015). Our results are directly relevant to these findings, as we show that expression of the ASIC1 gene is notably higher in TG neurons when compared to DRG. Moreover, we newly identified ASIC1 as differentially expressed compared to previous datasets (Manteniotis et al., 2013; Flegel et al., 2015), likely because its presence in oligodendrocytes (Zhang et al., 2014) would have obscured the neuron-specific difference in mixed tissues. This again highlights the importance of studying cell populations in isolation, as dilution effects can obscure crucial targets that might be used in clinic.

Besides genes linked to pain processing, our study also identified 15 Hox genes which are exclusively expressed in lumbar DRG neurons compared to TG. Hox genes are recognized for their key role as regulators of system development and cell fate specification during embryogenesis (Wellik, 2007; Mallo and Alonso, 2013; Philippidou and Dasen, 2013). Their continued presence in adult DRG is the most striking difference between the two types of ganglia: it can be detected at the level of whole TG and DRG (Manteniotis et al., 2013; Flegel et al., 2015; Kogelman et al., 2017), and similar anterior–posterior patterns are still discernible in adult CNS (Hutlet et al., 2016). In the brain, the expression of Hox genes after development has been linked to the regulation of key proteins that are involved in synapse formation and plasticity (Hutlet et al., 2016). Evidence is still sparse, but if it were to consolidate, Hox genes could represent an interesting exploratory drug target for some forms of chronic pain where changes in synaptic plasticity are well-recognized (Luo et al., 2014; Kuner and Flor, 2016). The absence of Hox genes from the TG is likely due to the distinctive pattern of homeobox gene expression during development (Hodge et al., 2007). We propose that, in the near future, these unique genes could also be used as “guides” in gene therapy, to either knockdown or overexpress genes in specific neurons during chronic pain states, as it has been proposed and already achieved in some specific cell types (Jang et al., 2007; Waehler et al., 2007; Clarke and McMillan, 2014).

Unveiling the specific molecular identities of sensory neurons is crucial for understanding the biology of these highly specialized cells under healthy conditions as well as for developing effective therapies in disease. This is the first report of a molecular comparison between enriched DRG and TG neurons. Our RNA-seq analyses show that despite unexpected similarity, these two populations of neurons have some unique molecular characteristics, as evinced by 63 differentially expressed genes. We are confident that our results will benefit the scientific community by providing easily searchable, neuron-specific information on DRG and TG expression patterns. Furthermore, good agreement between mouse neurons and human tissue suggests that our analyses may aid in the continued quest for better drugs against chronic pain conditions, including migraine.

## Materials and Methods

### Transgenic Mice

Avil-GFP mice [see gensat.org: STOCK Tg (Avil-EGFP) QD84Gsat/Mmucd for BAC expression levels] were bred in-house for several generations on a CD1 background. In all experiments, adult, age-matched (3–6 months) littermate controls from both genders were used. Mice were kept in a 12-h light–dark cycle, with food and water *ad libitum*. All experiments were performed in accordance with the UK Animals (Scientific Procedures) Act 1986 and Local Ethical Committee approval.

### Fluorescence-Activated Cell Sorting

Avil-GFP mice were deeply anesthetized with pentobarbital and transcardially perfused with 10 ml of PBS. TG (two ganglia/animal) or lumbar DRG (L1–L5, *n* = 10 lumbar ganglia/animal) were dissected out. Samples were then incubated in 3 mg/ml dispase, 0.1% collagenase and 200 U/ml DNase for 60 min at 37°C in a CO_2_ incubator, followed by gentle trituration in 1 ml of FACS buffer (5% trehalose, 0.05 mM APV, 15 mM HEPES in DPBS). PI (2.5 μg/ml) was added to the cell suspension so dead cells could be identified during sorting.

Following dissociation, neuronal cell isolation was performed using a BD FACS Aria II Cell Sorter at the NIHR BRC flow core facility at King’s College London (nozzle size 100 μM). Cell populations were gated on GFP+, PI- signal with the help of unstained and single-staining controls. For every experiment, cells from a non-transgenic control animal were used for compensation controls. Positive events (500/animal) were sorted directly into Qiagen RLT buffer and beta-mercaptoethanol and stored at -80°C until the entirety of the biological samples were collected and processed for RNA extraction using a RNeasy Micro kit (Qiagen) with minor modifications of the manufacturer’s protocol. Sample quality was confirmed on an Agilent Bioanalyzer (average RIN value of 8.75, min 7.6, max 9.9).

### Sequencing

RNA-seq was performed as previously described (Thakur et al., 2014). In brief, samples were sent for batch-controlled library preparation (SMARTer Ultra Low Input HV kit, 634820, Clontech) and sequencing at the High-Throughput Genomics Group at the Wellcome Trust Centre for Human Genetics at Oxford University. Following amplification, samples were multiplexed in replicate flow cells on an Illumina HiSeq 4000 platform. Reads were uniquely aligned to the mm10 genome using STAR (Dobin et al., 2013), yielding a median mapping percentage of 85.8% (min 80.7%, max 87.6%, standard mapping parameters, bar—outFilterMultimapNmax 1). Normalized gene expression values and differential gene expression data were generated using the cufflinks and DESeq2 algorithms (Trapnell et al., 2010; Love et al., 2014), respectively. We applied an FPKM expression cut-off, based on the underlying distribution of our cufflinks data: FPKM ≥ 1 in at least two of the replicates for each condition. Please note that expression cut-offs will be specific to each individual RNA-seq dataset (as FPKM values are modeled on a curve) and are necessarily somewhat arbitrary. Ours is quite liberal and will likely overestimate the presence of particular gene transcripts. Raw and processed data are available under GEO accession GSE100175.

### ELISA

CGRP (Calca) protein levels were measured from freshly extracted DRG and TG samples using a CGRP ELISA kit according to manufacturer’s instructions (Bertin Pharma, 589001). Briefly, the tissue was macerated with a hand-held homogenizer in 200 μl EIA buffer, and the resultant protein was bound by anti-CGRP antibody for 5 min at room temperature. Samples were washed five times and the signal developed with Ellman’s reagent and read on an ELISA plate reader (Molecular Devices, 410 nM). All samples, standards and negative controls were processed in duplicate. The resulting standard curve was linear as required (*y* = 0.004*x* + 0.0973, *R*^2^ = 0.98), and all samples fell within the limits of the standard samples.

### qRT-PCR

Animals were deeply anesthetized with pentobarbital and transcardially perfused with PBS. DRG (lumbar, L3–L6) and TG were collected and RNA was prepared with the RNeasy Micro kit (Qiagen). Total RNA (500 μg) was converted to cDNA using the SuperScript^®^ III Reverse Transcriptase kit (Thermo Fisher Scientific). Quantitative real-time PCR (qPCR) was performed in duplicate with a SYBR green master mix (Roche Diagnostics Limited) with the addition of the selected gene primer sequences (Sigma)—see below. ΔCts were calculated in relation to a house keeping gene (GAPDH). In all experiments, qPCR reactions were run in a Roche Lightcycler 480 PCR machine. Results were analyzed by the standard ΔΔCt method. All primers were checked for their efficiency and specificity beforehand.

Cgrp_F: CTGAGGGCTCTAGCTTGGAC;

Cgrp_R: TGCCAAAATGGGATTGGTGG

Hoxa7_F: CAGTTTCCGCATCTACCCCT;

Hoxa7_R: CGTCTGGTAGCGCGTGT

TrkA_F: GAAGAATGTGACGTGCTGGG;

TrkA_R: GAAGGAGACGCTGACTTGGA

Gapdh_F: GGTCCCAGCTTAGGTTCATCA;

Gapdh_R: CCAATACGGCCAAATCCGTTC

### Imaging

Avil-GFP mice were deeply anesthetized with pentobarbital and transcardially perfused with PBS followed by a 4% PFA solution. Tissue was dissected out (DRG and TG), post fixed for 2 h and placed in 30% sucrose solution and left overnight at 4°C. Samples were then embedded in OCT and cut on a cryostat (10 μm sections). Slides were incubated with blocking buffer (10% donkey serum, 0.02% Triton X-100 in PBS), for ∼6 h at room temperature. Sections were then incubated with anti-NeuN antibody (1:1000; Rabbit—Millipore) overnight at room temperature. Primary antibody was removed by washing with PBS, three times. Secondary antibody conjugated with Alexa 594 (Invitrogen) was diluted in the blocking buffer (1:1000), and slides were incubated for at least 2 h at room temperature. Slides were then mounted using Fluoromount-G^®^ medium (Southern Biotechnology) and allowed to dry for 48 h. Endogenous EGFP alongside with Alexa 594 from the sections were imaged using the tile imaging mode (mosaic function) in a Zeiss 710 LSM Axio Imager.Z2 Microscope, at 20×/0.8 DICII magnification. Images were processed (stitched and scale bars placed) using the Zen 2012—Blue Edition software (Zeiss).

### Statistics

For all the validation experiments, normality was confirmed by Shapiro–Wilk test. If samples showed a normal distribution, parametric tests were used, otherwise, samples were analyzed by a non-parametric test.

For the CGRP ELISA assay experiments, an independent samples *t*-test (two-tailed, heteroscedastic) was used. For the qPCR experiments, a Mann–Whitney non-parametric test was performed.

Statistical analyses were performed using Prism 5 software or SPSS.

## Author Contributions

DL, FD, and SM contributed to the design of the experiments and interpretation of the data. DL and FD performed the flow cytometry, molecular and cell biology experiments. FD performed the bioinformatics analyses. SM conceived the project. DL wrote the manuscript.

## Conflict of Interest Statement

The authors declare that the research was conducted in the absence of any commercial or financial relationships that could be construed as a potential conflict of interest.

## Abbreviations

Avil: advillin
AVRG: average
CGRP: calcitonin gene-related peptide
DRG: dorsal root ganglia
FACS: fluorescence-activated cell sorting
FPKM: Fragments Per Kilobase of transcript per Million mapped reads
GFP or EGFP: (enhanced) green fluorescent protein
Hox: homeobox
mRNA: messenger RNA
PI: propidium iodide
qPCR: quantitative polymerase chain reaction
RIN: RNA integrity number
RNA: ribonucleic acid
RNA-seq: RNA sequencing
TG: trigeminal ganglia.
