# Supplementary material for: The Molecular Fingerprint of Dorsal Root and Trigeminal Ganglion Neurons
Source: Front Mol Neurosci. 2017 Sep 26;10:304. doi: 10.3389/fnmol.2017.00304 (PMC5623188; doi:10.3389/fnmol.2017.00304)
Supplement: Supplementary file 4 [file Data_Sheet_1.docx]

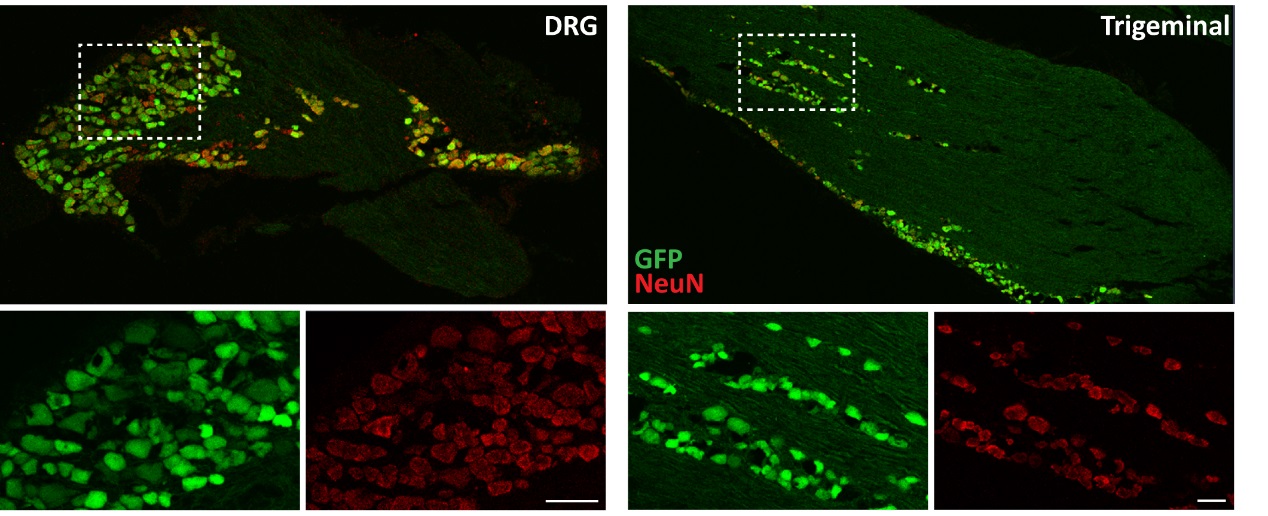


**Supplementary Figure 1: Avil is present in sensory neurons of the Avil-GFP transgenic mouse.** Representative images of DRG and TG extracted from the Avil-GFP mouse show that all green cells are positive for NeuN (in red), highlighting the expression of Avil in neurons only. Scale bar: 50 microns.


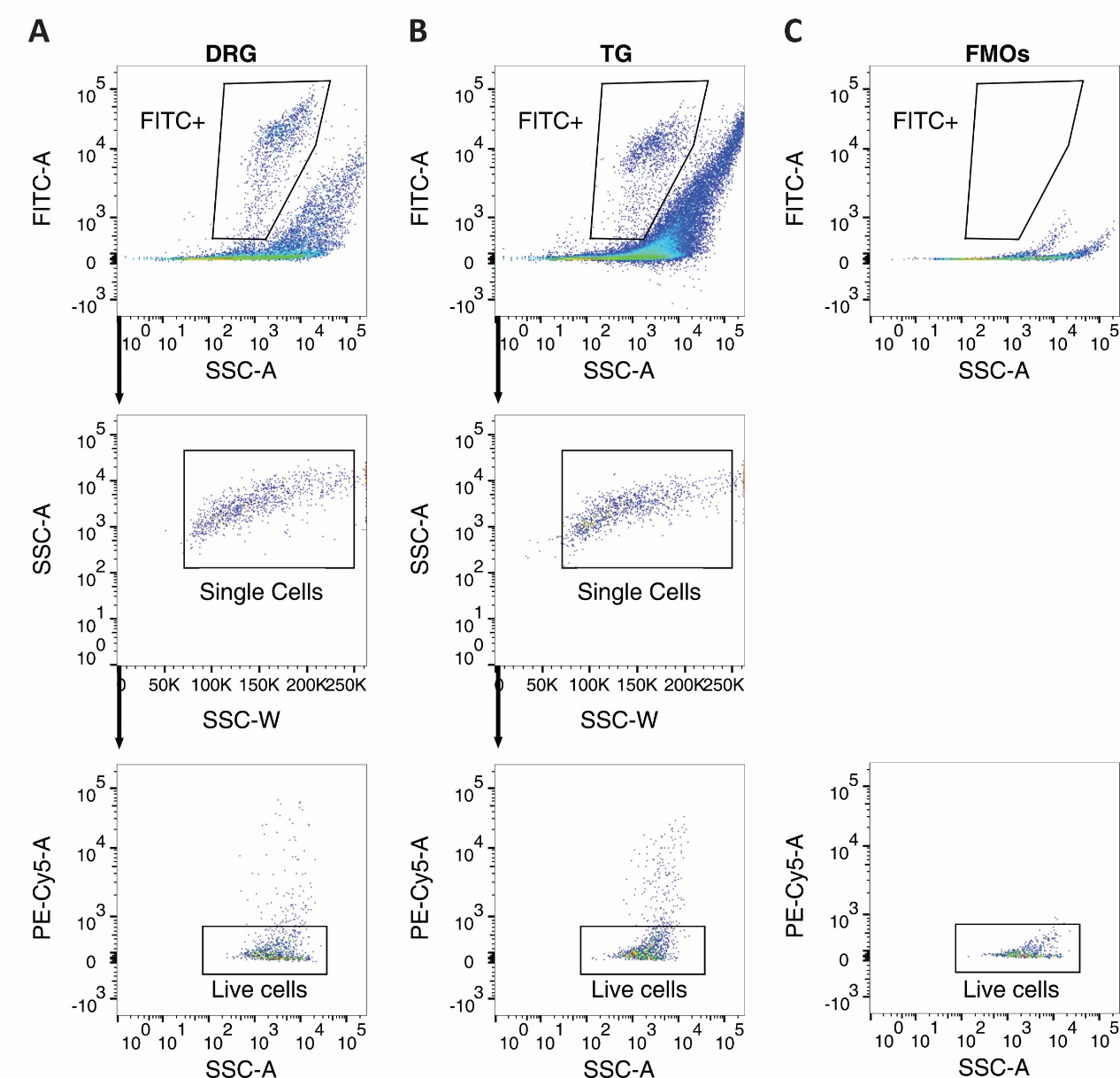


**Supplementary Figure 2: Detailed gating strategy for FACS.** **(A)** DRG or TG neurons were gated on GFP positive cells (FITC+), followed by side scatter area (SSC-A) vs. side scatter width (SCC-W) to distinguish single cells from clumps, and finally selected based on the live-dead stain propidium iodide (PE-Cy5 negative) **(B)**. The rightmost column **(C)** shows how gates were established using fluorescence minus one controls (FMOs). Please note that the machine can faithfully detect varying degrees of GFP fluorescence ranging from 10^3^-10^5^.


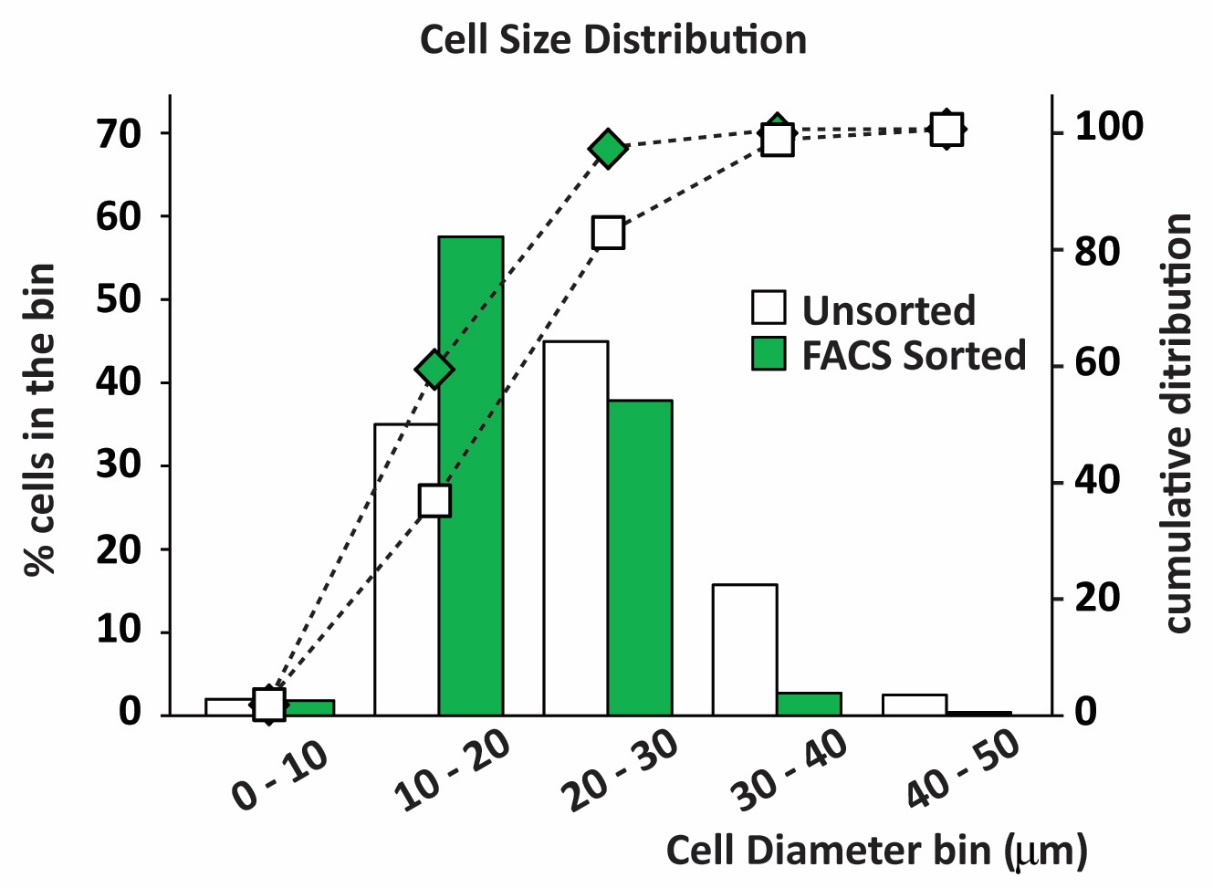


**Supplementary Figure 3: Cell size distribution of FACS sorted neuronal cells used in this study.** Graph illustrating cell size distribution present in the unsorted (white bars) and FACS sorted (green bars) preparations. Cumulative-sum chart shows the neuronal size distribution of FACS sorted cells (green diamonds) is slightly skewed to the left hand side, when compared to unsorted cells (white squares) reflecting a higher proportion of smaller diameter neurons present in the FACS preparation, as reflected in the RNA profile (see discussion). FACS data is representative of the quantification of 327 cells from the lumbar DRG of one animal. Data was plotted against previous published data (Unsorted) from our laboratory (Thakur et al., 2014).
